# This Statement Is Absolutely Untrue!

**Published:** 1919-12-13

**Authors:** 


### THE NURSES' CO-OPERATION.

### "This Statement is Absolutely Untrue!"

The Proprietors and the Editor of The Hospital separately have received letters from a firm of Solicitors, who write on behalf of Miss Maude McCullum, stating that in articles which appeared in The Hospital of November 15, November 22 and November 29, it was stated "that Miss "McCullum is improperly attempting to obtain by "reckless and dishonest methods the accumulated "funds of the Nurses' Co-operation for the purpose "of utilising them on behalf of the Professional "Union of Trained Nurses, which is about to be "formed."

**The Solicitors add, in their next paragraph: "This statement is absolutely untrue." We agree with them that the allegation that we made the statement alleged concerning Miss McCullum is "absolutely untrue."**

We assume the client on whose behalf the Solicitors write to be the lady referred to in our issue of November 22 as Miss Maud MacCallum. If this is so we apologise to Miss McCullum for having printed her name incorrectly.

The Solicitors call on us for a complete retractation of and full apology for the alleged statements, and threaten the Proprietors and the Editor with immediate proceedings claiming damages for libel, in default. They required a reply by Monday, December 8, the day on which their letters reached the Proprietors and the Editor.

**We have carefully gone through our issues of November 15 and 22 and find no such statements in them as are alleged. "The Hospital" of November 29 contains nothing on the subject.**

Having made no statements defamatory of Miss McCullum, we know nothing for which an apology is due from us to her. We therefore await the proceedings with which we are threatened, and which, if commenced, our solicitors are instructed to defend.

